# From competency to dormancy: a 3D model to study cancer cells and drug responsiveness

**DOI:** 10.1186/s12967-016-0798-8

**Published:** 2016-02-04

**Authors:** Josephine Y. Fang, Shih-Jye Tan, Yi-Chen Wu, Zhi Yang, Ba X. Hoang, Bo Han

**Affiliations:** Nimni-Cordoba Tissue Engineering and Drug Discovery Laboratory, Division of Plastic and Reconstructive Surgery, Departments of Surgery and Biomedical Engineering, Keck School of Medicine, University of Southern California, 1333 San Pablo St., BMT 302, Los Angeles, CA 90089 USA

**Keywords:** 3D cell culture, Microenvironment, Drug response, Dormancy, Competency

## Abstract

**Background:**

The heterogeneous and dynamic tumor microenvironment has significant impact on cancer cell proliferation, invasion, drug response, and is probably associated with entering dormancy and recurrence. However, these complex settings are hard to recapitulate in vitro.

**Methods:**

In this study, we mimic different restriction forces that tumor cells are exposed to using a physiologically relevant 3D model with tunable mechanical stiffness.

**Results:**

Breast cancer MDA-MB-231, colon cancer HCT-116 and pancreatic cancer CFPAC cells embedded in the stiffer gels exhibit a changed morphology and cluster formation, prolonged doubling time, and a slower metabolism rate, recapitulating the pathway from competency to dormancy. Altering environmental restriction allows them to re-enter and exit dormant conditions and change their sensitivities to drugs such as paclitaxol and gemcitabine. Cells surviving drug treatments can still regain competent growth and form tumors in vivo.

**Conclusion:**

We have successfully developed an in vitro 3D model to mimic the effects of matrix restriction on tumor cells and this high throughput model can be used to study tumor cellular functions and their drug responses in their different states. This all in one platform may aid effective drug development.

## Background

Solid tumors expose cells to a heterogeneous and complex extracellular matrix environment. Tumor phenotype are highly dependent on complex interactions with the surrounding cells and the ECM [1, 2]. Tumor ECM composition, fiber orientation, patterns of infiltration and volume have been used as independent clinical prognostic indicators several cancer types [3–5]. Matrix stiffness induced by type I collagen deposition and cross-linking has been shown previously to promote malignant transformation [6, 7]. Other extracellular matrix components such as laminin, fibronectin, tenascin have been observed in breast and primary small cell lung cancers in studies associated with cancer metastasis and drug resistance [8, 9].

In addition to ECM components, cells secreted enzymes such as lysyl oxidase (LOX) and matrix metalloproteinases (MMPs) also contribute to the establishment and maintenance of the pre-metastatic niche [10, 11]. LOX released from hypoxic tumor cells at the primary site are able to induce cross-linking of collagen and further increase matrix stiffness. The increased ECM stiffness could be a body defense mechanism to shield diseased cells from uncontrollable growth. On the other hand, the expression and activity of MMPs are enhanced in almost every type of human cancer and correlate with the progression of tumor stage, occurrence of invasion and metastasis, and mortality [12, 13]. In an in vivo study, the inoculated cancer cell proliferation was decreased in tumors generated from *Mmp9*-deficient mice compared to wild-type mice [14].

ECM signals in forms of ligand/receptor interaction and physical forces substantially influence not only tumor development, progression and drugs responses, but also may modulate entering and exiting from dormancy [2, 15, 16]. Increasing evidence suggests that tumor cells in a dormant state can escape chemotherapeutics, and recur from silence after years [17–19]. Currently, dormant cancer cell do not have specific biological markers and limited experiments models are available to study them [19]. Due to the lack of knowledge of dormant cancer cells, cancer treatments are difficult to decide upon and are often ineffective, or even detrimental for patients [20]. In preclinical studies, dormant tumor cells are difficult to isolate from humans or animals, and this dormant phenomena is difficult to recapitulate in the in vitro and in vivo experimental systems [21]. These technical challenges hindered the progress of anti-cancer drug development.

In our previous study [14, 15] of using 3D Col-Tgel model with the use of transglutaminase cross-linked gelatin, we have demonstrated that Col-Tgel is suitable for in vitro tumor bioengineering as well as in vivo xenograft tumor formation. We observed prolonged cell doubling time when cultured in 3D comparing to monolayer. Moreover, the matrix stiffness of Col-Tgel can be conveniently tuned by controlling the concentration of gelatin [22, 23]. Hence, a serial of different stiffness Col-Tgels were adopted in this study to examine the impact of the ECM restriction on the tumor cell proliferation, metabolism, and drug responses. The development of 3D in vitro models that consistently and reliably recapitulate the tumor phenotypes from competency to latency may provide insight into tumour behaviour and aid effective drug development.

## Methods

### Cell culture and cell embedment

Human breast carcinoma cell MDA MB-231, pancreatic cancer cell line CFPAC-1, and colorectal carcinoma cell line HCT-116 cells (ATCC, American Type Cell Collection, VA) were cultured in high glucose Dulbecco’s modified Eagle medium, Iscove’s Modified Dulbecco’s Medium, or McCoy5a modified medium (DMEM, IMDM, and McCoy5a, Corning, VA) with 10 % (v/v) fetal bovine serum (FBS, Hyclone, ThermoScientific) and 1 % (v/v) penicillin- streptomycin (PS, Corning, VA) respectively in a humidified atmosphere of 5 % CO2, at 37 °C. Culture medium was changed every 2–3 days and cells were subcultured when cells reached 80 % confluent.

The cell 3D embedment culture was initiated by detaching cells with 0.25 % trypsin in HBSS (Corning, VA). Required cells number (2 × 10^6^ cells/mL) was aliquoted to individual micro- centrifugal tubes, and pelleted by centrifugation. Cells were dispersed evenly in soft, medium and stiff Col-Tgel (101Bio, CA) following manufacture protocol. The gel stiffness ranges from 0.8–50 kPa. A droplet with 20 μL of cell-gel mixture was casted on each well of 48-well suspension cell culture plate and formed a half-dome-shape on the non-treated tissue culture well surface. Cell type specific medium with volume of 500 µL was added into each well to submerge the constructs after the gel solidified through enzymatic corsslinking at 37 °C for 1 h. Medium was changed every 2–3 days.

### Cell proliferation and viability assay

Cell counting, performed to determine the cell growth and doubling times, was deduced from two time points (6 and 12 days after seeding) of cell-counting. Briefly, at the end of incubation, 3D constructs were washed with cold PBS three times and digested with 200 µL of 0.25 % trypsin in HBSS for 4 hours with rocking to release resided cells. After neutralize the enzyme activity with complete medium, the digested cells were collected and further diluted to 10 mL for cell count (Cell and Particle Counter, Beckman Coulter, CA). Experiments were performed triplicates and data represented mean ± S.D. in the graph. Particle size gated at 9.49 µm to exclude cell debris.

The total viability of Col-Tgel encapsulated cells was quantified using a by Cell-counting kit-8 (CCK-8, Dojindo Molecular Technologies, MD). Cell constructs were incubated in 300 µL of CCK-8 working solution for 4 h and the absorbance at 450 nm was measured with a plate reader (Molecular Device, CA). Medium without cells was served as control. In addition, the viable cells were visualized with MTT (3-(4, 5-dimethylthiazol-2-yl)-2, 5-diphenyltetrazolium bromide, Sigma-Aldrich, MO) staining. The cell constructs were incubated with MTT working solution (filtered MTT 5 mg/mL in culture medium, 1:10) for 4 h at 37 °C and imaged were captured with light microscope counted digital camera (Nikon, Japan).

### Alteration of 3D environmental conditions during cell culture

In order to simulate the alteration of the matrix conditions during tumor progression, MDA-MB-231 cells were first released from their original 3D culture conditions at day 6 and re-embedded into their counter gel conditions, e.g., from stiff to soft or vice versa. Cells were further cultured for an additional 6 days before cell counting.

Exogenous enzyme digestion was performed to simulate matrix remodeling by proteases derived from paracrine secretion of neighboring cells. The cell–matrix constructs were treated with either 5 unit/mL type-2 collagenase (Sigma-Aldrich, MO) for 1 h or 0.025 % trypsin in HBSS (Corning, VA) for 3 min. Enzymes were inactivated by washing with culture medium three times. These digestion conditions were pre-determined by cell type specific doses that induced unnoticeable destruction of constructs. Treated constructs were cultured for additional 6 days before cell counting.

### Immunocytochemistry

Cytoskeletons were stained for F-actin to monitor cell morphology change in 3D culture. Briefly, the cell/gel constructs were fixed at predetermined time points with 10 % phosphate buffered formalin for 10 min, washed with Tris-buffered saline and Tween-20 (TBST) for three times, and stained with 30 nM rhodamine phalloidin and 30 nM DAPI dihydrochloride in the dark at room temperature (Life Technologies, NY). The live and dead cells populations were distinguished by directly staining with LIVE/DEAD Viability/Cytotoxicity kit for mammalian cells (Life Technologies, NY) followed by manufacture protocol. The stained constructs were observed and recorded under EVOS fluorescence microscope (Life Technologies, NY).

### Gelatinolytic zymographic assay

The activity of cell layer matrix metalloproteinases was detected by gelatin-based zymography. Cell embedded constructs were cultured for 5 days before transferred into microtubes for homogenizing with 0.25 % Triton X-100. After repeated freeze/thaw and centrifugation, supernatant was collected for zymograph assay on a Ready Gel Zymogram Gel (Bio-Rad, CA) at 90 V for 3 h. After a brief wash with water, the gel was incubated with 2.5 % (v/v) Triton X-100 and subsequently replaced with a developing buffer containing 50 mM Tris, 5 mM CaCl_2_, and 200 mM NaCl pH 7.5 at 37 °C for 24 h. The gelatinolytic activities were visualized by staining with Coomassie blue solution (62.5 % ethanol, 25 % acetic acid and 0.125 % Coommasie blue R250 (Bio-Rad, CA) and excessive staining removed by the de-staining solution (30 % methanol and 1 % formic acid).

### Drug sensitivity assay in using cells in competent and dormant states

To estimate the drug response under cell proliferation fast- and slow-growing conditions, cells were first cultured in three different stiffness Col-Tgels for 7 days to induce tumoroid formation. Gemcitabine (APP Pharmaceuticals, LLC) was reconstituted in 0.9 % sodium chloride and further diluted into experimental conditions. Paclitaxel (TEVA Pharmaceuticals, Inc.) was diluted into experimental concentrations with 10 % FBS DMEM. Cells were treated with either 6.25 or 25 µM of drugs (final concentration) for 72 h. One subset of constructs were assayed by cell counting and live/dead cell staining right after drug withdrawal without recovery; and another set were cultured in fresh medium for additional 4 days for recovery before analysis. The schematic description was depicted in Fig. 3b.

### In vitro re-growth of drug treated cells

To verify the tumor formation potential of drug treated survived cells, MDA-MB-231 or CFPAC-1 cells were first embedded in dormant conditions (also defined as high stiffness matrix) to receive 72 h of drug treatment followed by 96 recovery. For MDA-MB-231, drug doses were 2.5 µM for paclitaxel or 12.5 µM for gemcitabine respectively. For CFPAC-1 cells, drug doses were 12.5 µM for paclitaxel or 12.5 µM for gemcitabine respectively. Cells were released from gel and re-embedded in low, medium or high stiffness gel matrices. The cell counting was performed after 6 days of culture and compared with cells without drug treatment.

### In vivo tumor formation of drug treated cells

MDA-MB-231 cells that survived drug treatment were implanted in athymic nude mice for xenograft tumor formation. Survived drug treatment cells were released from high stiffness gel construct and re-encapsulated in soft Col-Tgel for subcutaneous delivery. Each athymic nude mouse (male, Charles River) received a injection of 100 µl of Col-Tgel (101 Bio, Palo Alto, CA) with 10^6^ cells on each side of the caudal frank. Each group included six inoculate sites. Tumor sizes were measured twice a week and tumors were harvested at day 28 and processed for histological analysis. All procedures were performed in accordance to Institutional Guidelines and Protocols that approved by University of Southern California Institutional Animal Use and Care Committee (USC IACUC). Harvested tumors were processed for fixation, dehydration, paraffin embedding and sectioning. Specimens were stained with Harris’ hematoxylin and counter stained with eosin.

### Statistical analysis

Experiment results were analyzed with rank transformation linear regression method by SAS (SAS institute Inc.). Due to the small amount sample size, experiment data were converted to rank transformation and analyzed correlation and regression of each study group with rank transformation by PROC GLM. Since in vitro sample size were the same, we choose Tukey’s Studentized Range (HSD) Test for the same sample size pairwise comparison (**p < 0.01, and *p < 0.05).

## Results

### Cancer cell proliferation rates, morphologies, and metabolic rates are cell type and micro- environment dependent

**Fig. 1:**
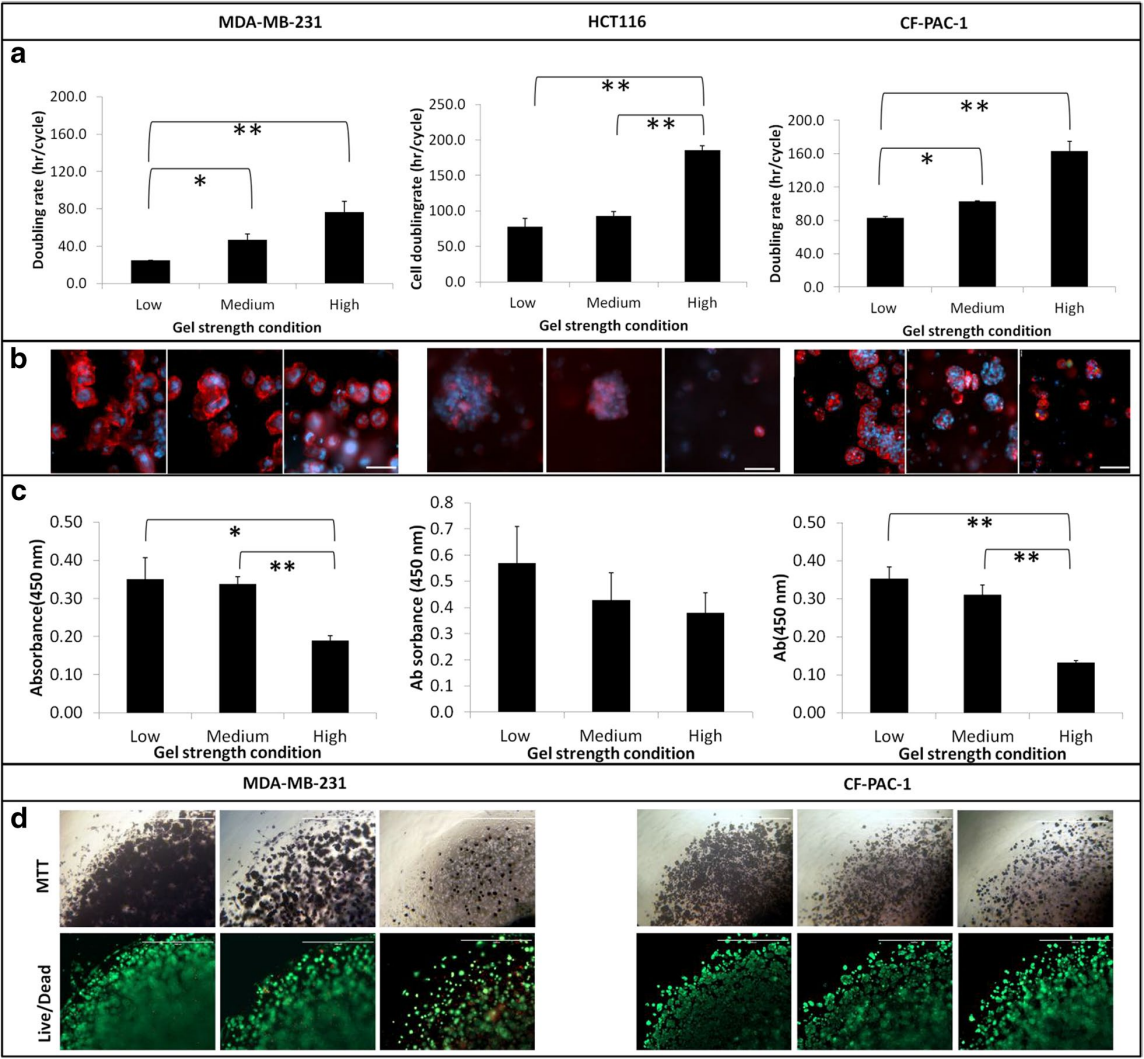
Changes in matrix stiffness regulate cell doubling time, morphology and cellular activity. Three different types of cancer cell lines MDA-MB-231, HCT-116 and CFPAC-1 were embedding in Col-Tgel with the stiffness range of 0.8–50 kPa. Cell doubling time prolonged with the increase of matrix stiffness (**a)** cells tend to form big clusters in soft gel but compact clusters in hard gel (**b**), *scale bar* = 40 µm); and total reductive activity decreased with the increase of gel stiffness in different origins of carcinomas (CCK-8) (C). The location of active cells inside gel was shown in (**d**, *top*), The MTT unstained cells (*white spots*) were verified as dormant cells due to lack of dead cell staining (Live/Dead staining, Live: *green*, Dead: *Red*) (**d**, *bottom*), *scale bar* = 1000 µm. Statistical significance was performed by Tukey’s test comparing with low condition, ** representing p < 0.001.* representing p < 0.01

Tumor cells sense extracellular matrix rigidity through bidirectional interaction with the surrounding ECM proteins and respond accordingly. In order to study the impact of matrix restriction on cell proliferation, we tuned gel stiffness by altering gel concentration as previously described [22, 23]. Three carcinoma cell lines, breast adenocarcinoma cells (MDA-MD-231), pancreatic ductal adenocarcinoma cells (CFPAC-1) and colorectal carcinoma (HCT-116) were selected and embedded in gels with the same initial seeding density. After 6 days of culture, cell number, cellular activity and morphology displayed significant differences among cells that embedded in different stiffness gels (Fig. 1). In comparing to 2D, 3D embedded cells had relatively longer doubling time and it extended even longer with an increment of gel strength. The doubling time in 3D culture ranged from 25 to 77 h for MDA-MD-231, 78–186 h for HCT-116 cells, and 83–163 h for CFPAC-1 cells in different stiffness gels. This observation is coherent with other reports that cell proliferation in the 3D hydrogel was relative slower in comparison to monolayer cell cultures [24, 25]. The expression of the proliferation marker, Ki67 is additional evidence to support this observation. The Ki67 protein expression in MDA-MB-231 cells reduced as ascending gel strength in 3D culture (data not shown). These observations indicated that the cell proliferation state could be altered by the gel strength.

Apart from the variations in doubling time, we also observed distinctive cell morphologies and cluster formation profiles among different gel conditions (Fig. 1b). After 6 days of culture, MDA-MB-231 cells in soft or medium gels are prone to form irregular and spiky contour clusters and some clusters further aggregated into mesh-like network with elongating actin filaments (Fig. 1b). Under similar conditions, HCT-116 and CFPAC-1 showed similar inclination to generate smooth contour bubble-like clusters. These big clusters could contain more than a hundred cells in each assembly. On the contrary, three cell types all presented small round compact clusters with less than ten cells or stayed as individual cell in the stiff gel condition. In general, the cluster formation, known as tumoroids, tended to occur in the soft gel condition.

In addition to cell proliferation, the overall metabolism rate of each construct was quantified in terms of reduction potential by using indicative molecule, 2-(2-methoxy-4-nitrophenyl)-3-(4-nitrophenyl)-5-(2,4-disulfophenyl)-2H tetrazolium (CCK-8), a water soluble form of tetrazolium. CCK-8 results suggested that the overall cell metabolism rate or reduction potential was sequentially decreased as the gel strength increased. Three cell types all demonstrated the same propensity in the tested range of gel strength (Fig. 1c). To visualize the location and distribution of highly active cells in situ, we identified cell metabolism state using another indicative molecule 3-(4, 5-dimethylthiazol-2-yl)-2, 5-diphenyltetrazolium bromide (MTT). MTT was taken up into cells by endocytosis into cells and converted to purple insoluble crystals by active dehydrogenases and reductases [26, 27]. The intensity of purple insoluble crystals could indicate the location of active metabolic cells (Fig. 1d, upper column). Cells in the soft gel condition had large quantity of viable cells that scattered evenly both around gel edge and at gel center. Under this condition, the majority of cells formed large clusters and their color appeared to be darker than those single ones after MTT staining. Based on these observations, we defined these highly proliferative cells as competent cells and their corresponding gel conditions that supported highly cell proliferation and cluster formation as proliferation competent gel conditions. With the increment of gel stiffness, cell cluster number and cluster size were reduced. When gel condition reached the stiffest investigative range, trivial single cells showed MTT dye retention in the cytoplasm or membrane surface. Primarily, only those cluster-forming-cells exhibited dark purple. Most single cells remained transparent (white spots). This observation suggest that cancer cells metabolism rates in terms of MTT uptake and reduction are strongly modulated by environmental cues and the gel restriction may be one parameter to determine the cellular activity. Within experimental gel conditions, the reagent such as MTT dye was capable of penetrating to the center of all gel conditions without geometry or porosity hindrance. To validate the viability of these transparent cells that were not MTT stained, we applied trypan blue exclusion staining to the gel released cells. These cells were viable with integrative cell membrane (released cells < 5 ± 2.8 % stained positive with trypan blue). In addition, these cells were also stained in situ with calcein-AM and ethidium homodimer-1 to identify the live/dead cell distribution in the 3D gel (Fig. 1d lower column). The overall dead cells in gel constructs were less (<2 %) and frequently occurred in the center of the gel. This result indicated these non-MTT staining cells were not dead cells whereas some of the non-MTT staining cells stained positive for the calcein-AM (green). Noticeably, we observed that approximately half of the cell population in high gel conditions were neither stained by calcein-AM nor ethidium homodimer-1. Hence, we defined these cells as according to the gel condition that was able to induce a large population of slow proliferation or dormant cell, as dormant gel condition.

### Cancer cells exit dormancy by modulating the property of the matrix

**Fig. 2:**
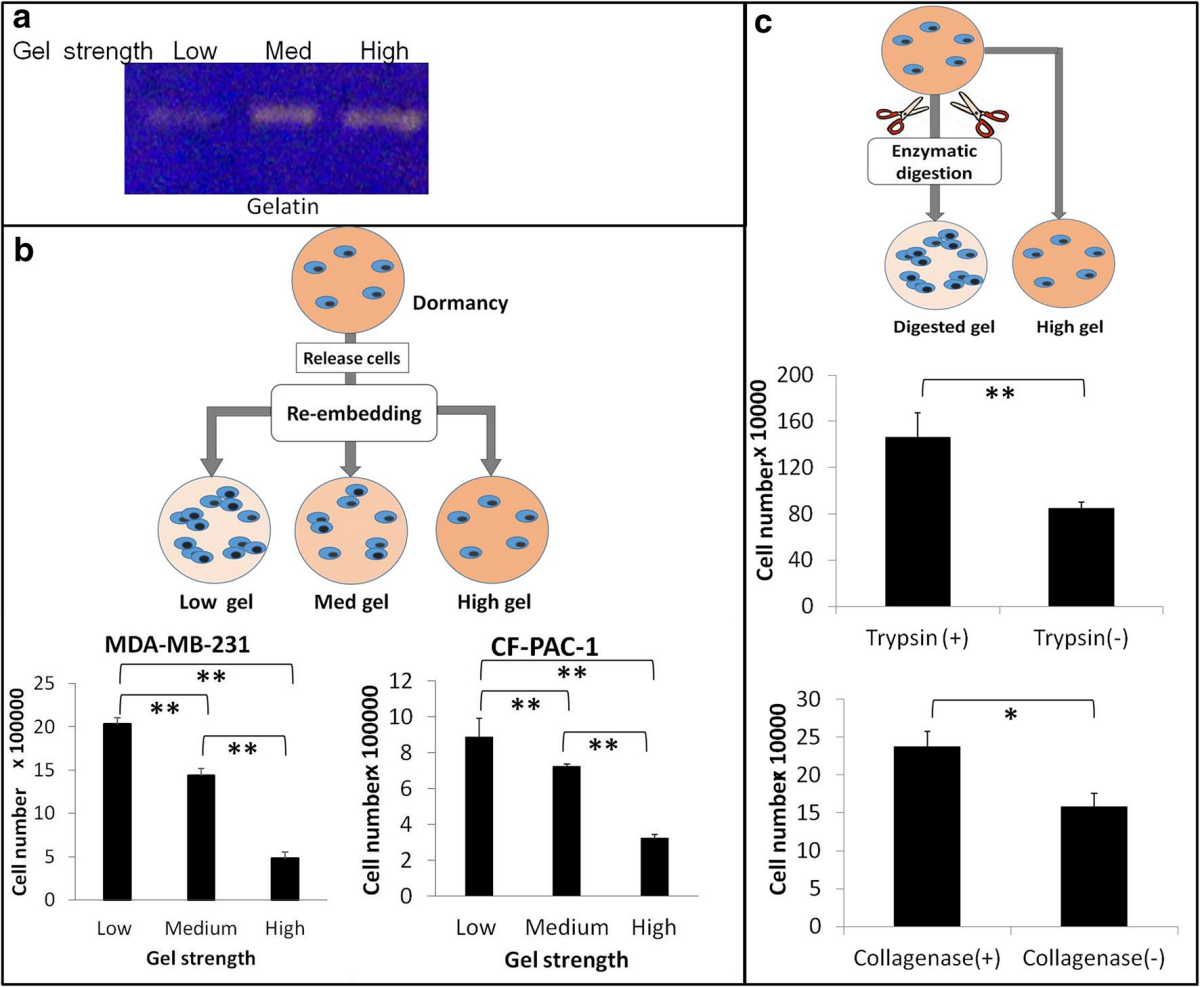
Positive and passive changes of environmental stiffness regulate cell proliferation rate. **a** MDA-MB-231 expressed more MMPs to cope with increased mechanical stress. Enzyme secretion from MDA-MB-231 cells embedded in different gels for 7 days and analyzed with zymography; **b** the cell states of dormancy and competency could be altered by re-embedding cells into different stiffness gels; **c** exogenous enzymatic digestion of gel matrix changed MDA-MB-231 proliferation rate in situ. Tukey test significance * represented p < 0.05 and ** represented p < 0.01

Tumor progression is a dynamic process. While cells sensing matrix generated signals, they also expressed specific type and quantity of enzymes to modify their surroundings. In the gelatinolytic and caseins zymography assays, we observed that gelatinase activity in MDA-MB-231cells was enhanced with the increase of gel stiffness while casein activity decreased (Fig. 2a). This phenomenon may imply that cells secrete matrix proteases to remodel or escape from their environment when they sense stress. To answer whether matrix remodeling process could impact the cell proliferation rate, we released the cells from either proliferative competent or dormant conditions and re-embedded them in switched gel conditions. No matter what original gel stiffness cells resided in, they were able to follow the same trend that soft gel, and could support fast cell growths while stiff gel could hinder cell growths (Fig. 2b). Additionally, we applied exogenous enzymes, trypsin and collagenase to simulate environmental enzymatic actions exerted through autocrine or paracrine fashions. After brief digestion of 3D gels without disrupting the gel structures, the total cell number together with quantity of clusters in the digested gels were increased compared with non-enzyme treated counterparts after another 6 days culture (Fig. 2c). The same phenomenon was also observed in the CFPAC-1 cells (data not shown). After the treatment with collagenase, cells embedded in high stiffness gels underwent fast doubling cycle and increased cell number significantly (p < 0.05). These observation suggested that the cancer cell proliferation rate could be altered by environmental collagen stiffness; the dynamic remodeling of ECM environment could be achieved through ECM deposition/degradation and/or ECM modification by crosslinking/dissociation [28, 29]. This dynamic ECM remodeling could switch cancer cell from dormant to proliferative or vice versa.

### Cancer cells in competent conditions are more sensitive to chemotherapeutics

**Fig. 3:**
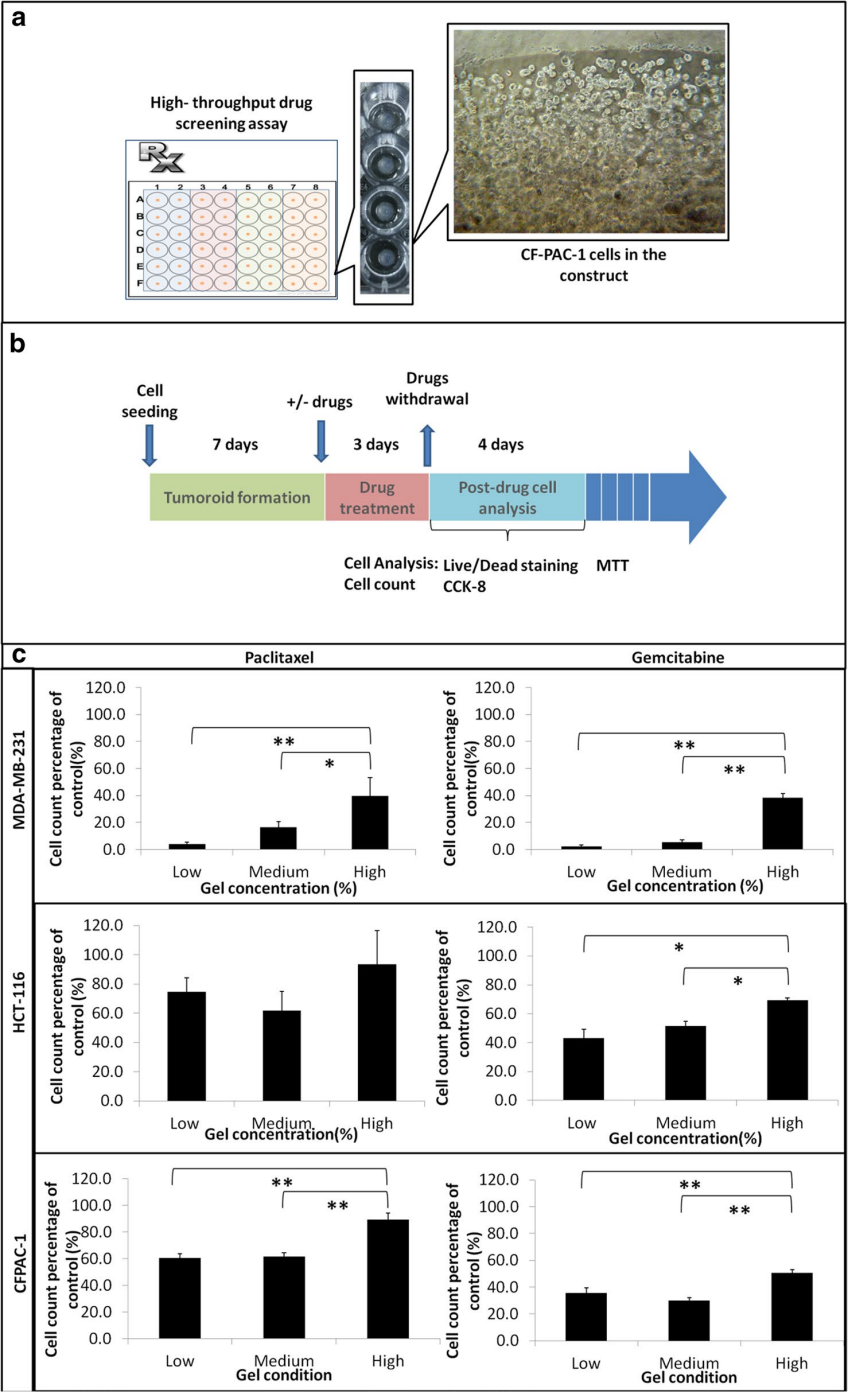
Matrix stiffness modulates cell responses to chemo-agents. **a** High throughput drug test models was setup in 48 well plate; **b** a schematic illustration of drug treatment and cell analysis schedule; **c** cells were more sensitive to chemo agent at competent state and less sensitive at dormant state. Grafts are plotted based on the percentage of survived cell counts over non-treated control after 72 h of 25 µM paclitaxel and 25 μM gemcitabine treatment and 96 h recovery. The statistical significance was performed by Tukey test (p < 0.05, *)

We then implemented the matrix strength variation into 3D model for drug response test. A high throughput drug test model was illustrated (Fig. 3a). In each well of 48-well plate, a single constructs was generated to receive designed drug treatment. Drug treatment procedure was outlined in Fig. 3b. Each of MDA-MB-231, HCT-116, or CFPAC-1 cells were cultured in three gel conditions respectively for 7 days to allow spontaneous cluster formation before receiving either paclitaxel or gemcitabine treatment for 72 h. Cell count and in situ cell staining for live/dead cells were performed on day 1 and 4 post-drug withdrawals. Paclitaxel is the first-line chemotherapeutic for breast cancer patients. Under proliferation competent conditions (soft gel), only 3.9 % of MDA-MB-231 cells survived paclitaxel treatment. The survival rate increased to 16.6 % when gel stiffness increased to medium condition. Total of 39.8 % cells survived when MDA-MB-231 cells resided in a dormant condition (stiff gel) (Fig. 3c, left panel, MDA-MB-231). Paclitaxel showed less effective for HCT-116 cells and CFPAC-1 cells in the 3D model, since more colon cancer cells and pancreatic cancer cells survived paclitaxel treatment even at their competent states (74.5 and 60.6 %, respectively). When these cells were under dormant condition, the survival rate increased to 93.5 % for HCT-116 cells and 89.3 % for CFPAC-1 cells (Fig. 3c, left panel, HCT-116 and CPFAC-1). These observations suggested that cancer cell drug sensitivity was both cell type and cellular environmental dependent.

To confirm the trend of the drug resistance, a second chemo-drug gemcitabine was tested in the same 3D settings. Gemcitabine is a clinically effective drug and is the first choice for breast, colon, and pancreatic cancers in chemotherapy. In cell proliferation competent gel conditions (soft gel), 2.4 % of MDA-MB-231 cells, 43.2 % of HCT-116 cells, and 35.5 % CF-PAC-1 cells were able to survive after gemcitabine treatment. When more cells enter the dormant state with increasing matrix restriction, the survival rate increased to 38.5 % for MDA-MB-231 cells, 69.5 % for HCT-116 cells, and 50.8 % in CF-PAC-1 cells. The ineffectiveness of gemcitabine was remarkably presented in both HCT-116 cells and CF-PAC-1 cells (Fig. 3c, right panel). In the intra group comparisons, both chemotherapeutics were more effective towards cells in their proliferation competent states than that at dormant states.

### Surviving cells after drug selection retain their tumor formation potential in vitro and vivo models

**Fig. 4:**
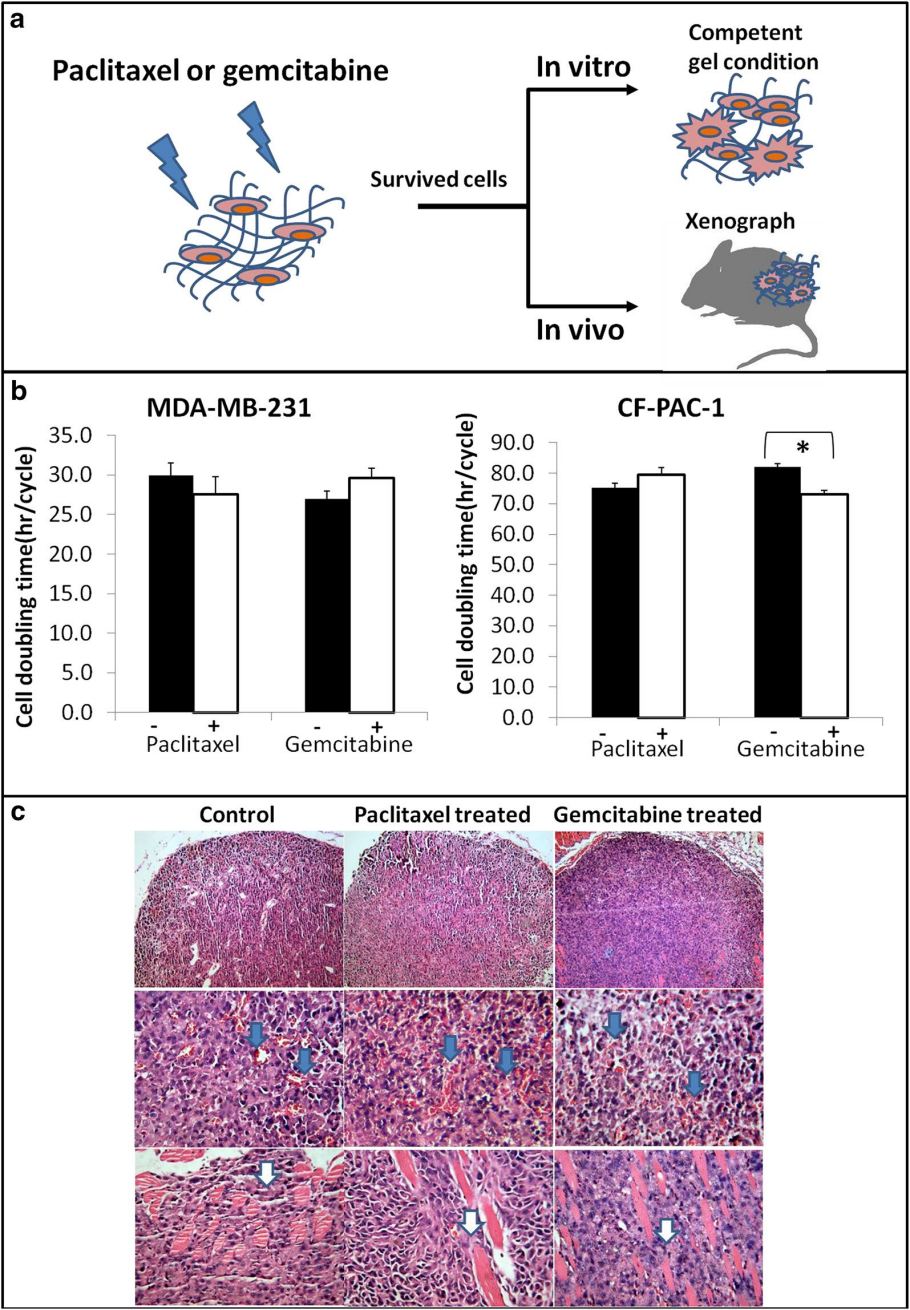
Cells survived drug treatments in stiff gel conductions are still capable of forming tumoriods in vitro and xenograft tumor in vivo. **a** A schematic illustration of the experimental procedure; **b** surviving cells have similar proliferation potentials when placed in competent conditions; Doubling time of survived MDA-MB-231 and CF-PAC-1 were similar compared to non treated control; **c** both drugs treated MDA-MB-231 cells were able to develop mature tumor in vivo *after* 4-week of inoculation in athymic nude mice (n = 6); *Blue arrows* showing angiogenesis occurrence in the tumors and *white arrow* revealing tumor cell muscle infiltration

Both paclitaxel and gemcitabine displayed reduced drug efficacy in a cell slow proliferation or dormant conditions. We intended to confirm whether these drug treated surviving cells still displayed tumorigenicity in vitro and in vivo. The procedure is illustrated in Fig. 4a. Paclitaxel or gemcitabine treated MDA-MB-231 cells and CFPAC-1 cells were released from stiff gel and re-embedded into a competent gel condition for 6 days. Both drug treated MDA-MB-231 cells and CFPAC-1 cells were able to competently proliferate in the new environment and displayed similar cell doubling rates as non-drug treated counterparts except the gemcitabine-treated CF-PAC-1 (Fig. 4b). The doubling time of the gemcitabine-treated CFPAC-1 was relatively slower than those without treatment (p < 0.05). However, cells released from competent conditions after drug treatment failed to survive (data not shown). When we inoculated the drug treatment surviving cells in animals for xenograft tumor formation, we observed mature tumor formation after 4 weeks in most cases (Fig. 4c). MDA-MB-231 cells treated with paclitaxel t in a high stiffness gel had similar tumor formation rates as non-drug treated counterparts (Table 1). Histologically, cancer cell density, vessels formation, and muscle infiltration were similar in treated or untreated cells (Fig. 4c). For gemcitabine treated cells, tumor formation rates were lower than for non-treated cells, but these tumors remain histologically similar (Table 1). Both in vitro and in vivo results suggested that dormant tumor cells receiving one round of drug treatment still maintained their tumor formation potential and were able generate mature tumors after drug withdrawal.

**Table 1:** Tumor formation rat in rat xenograft model

	Control (%)	Post-gemcitabine treatment (%)	Post-paclitaxel treatment (%)
Tumor formation rat	6/6 (100)	4/6 (66.67)	6/6 (100)

MDA-MB-231 cancer cells surviving in high density gel after gemcitabine or paclitaxel treatment were released and transplanted in athymic nude rat for 28 days

## 3D matrix stiffness modulates apoptosis process and redox status

**Fig. 5:**
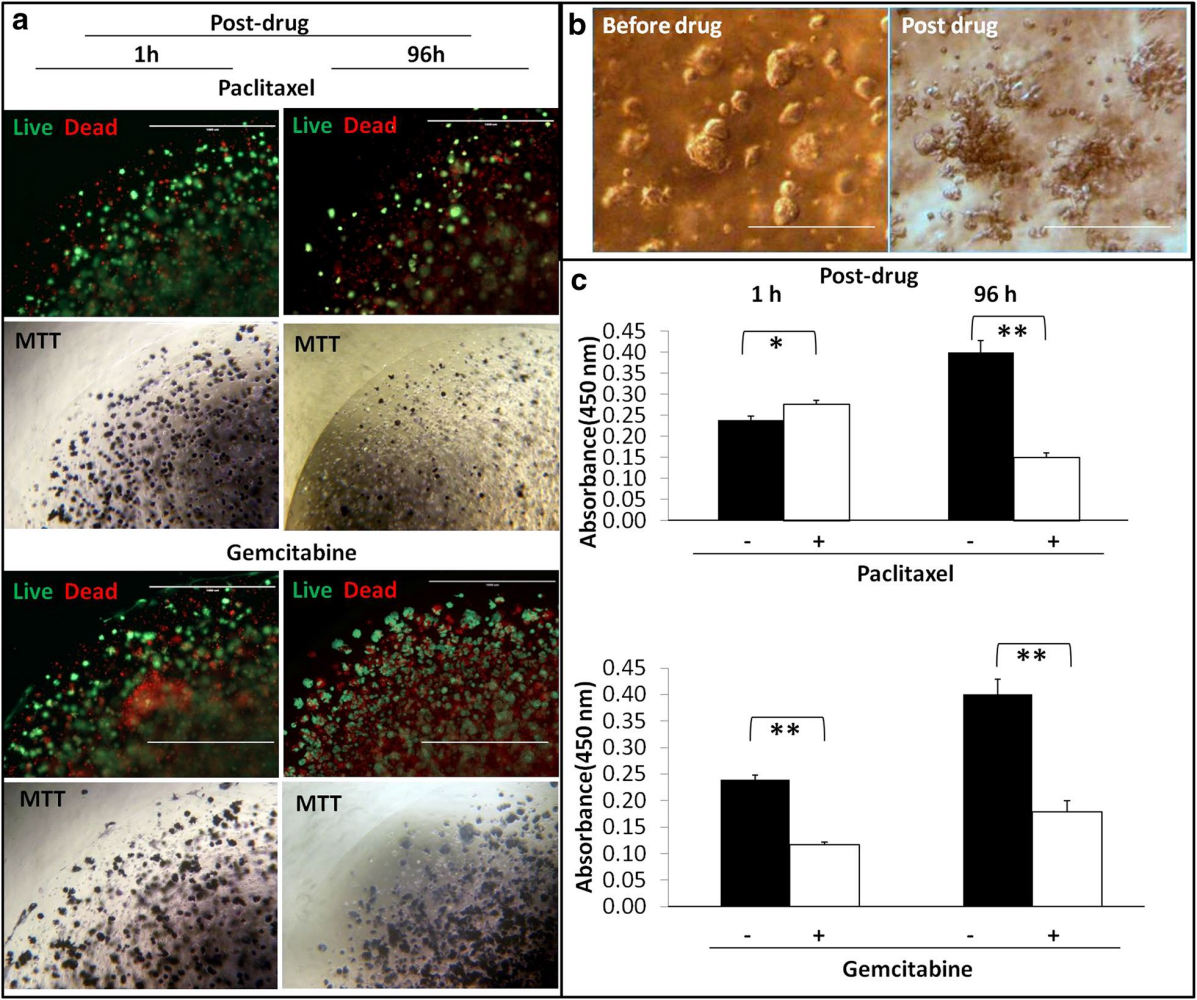
Matrix stiffness regulates drug induced apoptosis and redox status. **a** Live/dead cell staining of MDA-MB-231 cells post-72-hour drug treatment; Cells were allowed to recover in fresh medium for 1 h (*left*) and 96 h (*right*), more dead cells and less live cells in 96 h recovery, *scale bar* = 1000 µm; **b** MDA-MB-231 cell clusters dissembled after paclitaxel treatment, *scale bar* = 100 µm). **c** The redox status (CCK-8 reduction) of MDA-MB-231 changed post drug treatment, 1 vs. 96 h of recovery, *p < 0.05, **p < 0.01

Matrix stiffness induced tumor cells resistant to the conventional chemotherapeutic drugs. We therefore investigated whether the stiffness of the cancer cell niche regulates the susceptibility of MDA-MD-213 and CF-PAC-1 cells to chemotherapy-induced apoptosis. MDA-MB-231 cells were stained with live/dead markers in situ at both 1 and 96 h post-drug treatment time points in dormant gel conditions. The population of dead cells (ethidium homodimer-1 positive, red) was progressive increased from post drug hour 0–9 while live cells (Calcein-AM positive, green) gradually decreased (Fig. 5a, live/dead). This delayed apoptosis was exhibited in both paclitaxel and gemcitabine treatments. MTT in situ stains for viable cells also demonstrated delayed losing dark purple cell populations at 96 h post-drug treatment for both cell lines under two drug treatments (Fig. 5a, MTT). As shown in Fig. 5b, MDA-MB-231 cells formed abundant clusters at proliferation competent gel condition; after drug treatment, cell clusters dissembled into smaller aggregates or individual cells (Fig. 5b). Cell and cluster counting (particle size) revealed that the percentage of survived MDA-MB-231 dropped more significantly than CFPAC-1 cells after 96 h of post drug recovery period (data not shown). These results suggest the matrix stiffness induced slow growing delayed drug induced the apoptosis. The patterns of apoptosis were cell type and drug specific.

MTT, XTT (2,3-bis-(2-methoxy-4-nitro-5-sulfophenyl)-2H-tetrazolium-5-carboxanilide), and WST (2-(2-methoxy-4-nitrophenyl)-3-(4-nitrophenyl)-5-(2,4-disulfophenyl)-2H-tetrazolium, monosodium salt) are commonly used in quantifying cell viability by reduction of pyridine nucleotide cofactor, NADH [30]. Noticeably, we observed redox in terms of CCK-8 reading of the whole gel/cell construct was elevated instead of decreased after 72 h of paclitaxel treatment (p < 0.05) (Fig. 5c, paclitaxel, 1 h). The cell viability assays (MTT, XTT and WST) are based on all types of reductases in the cell respiratory cascade to reduce tetrazolium to formazan. When paclitaxel-induced oxidative stress increased in cells, coupled anti-oxidative signals may turn on to help cells escape drug damage and cell redox status changes [31, 32]. After an additional 96 h of recovery, the absorbance of CCK-8 dropped down to lower than control. This result confirmed the phenomenon of delayed cell apoptosis. However, gemcitabine did not show this delayed apoptosis and the trend was coherent at both time points. It implied that gemcitabine triggers a divergent cytotoxicity mechanism from that of paclitaxel (Fig. 5c. Gemcitabine).

## Discussion

In this study, we demonstrated that the stiffness of the 3D matrix profoundly alters the phenotype and behavior of three different cancer cells in vitro. Cell proliferation and metabolism rate were all decreased when the ECM restriction (stiffness) increased. Increasing evidence reveals that tumor cell genotype and phenotype are highly dependent on complex interactions with the surrounding cells and the ECM [1, 2]. However, to recapitulate these phenomena and assemble all environmental factors in vitro is not an easy task. Until now, 2D tumor cell cultures which seldom mimic the in vivo environment are still used routinely for conducting biochemical and drug tests. Increasingly more 3D models have been developed and applied in oncology [33], but lacking of physiological relevance limit their application and sometimes misinterpret the results. Biocompatible materials such as agarose, methylcellulose, PMMA, or PEG are structurally suitable to provide support for in vitro tumor study, but their structures lack cell adhesion and enzyme cleavage sites to correlate in vivo tissue surrounding [34, 35]. Cells grown on different stiffness surfaces coated with ECM [36] or synthetic polymer [16] have been used to study material stiffness impact on cellular functions. These models also have limitations since at cellular scale, the surrounding fluid and the extracellular matrix exert stresses on cells in an omnidirectional manner [37]. While cells on matrix coated surfaces are still exposed to two-directional forces they also lack spatial restriction for their expansion. This may be the reason behind the discrepancy of our results from others. When cells were grown on mechanically tunable polyacrylamide gels, it has been found that hepatocellular carcinoma [16] and glioblastoma [38] cell proliferation rates were promoted by increasing of the matrix stiffness. Noticeably, matrix stiffness is always coupled with other matrix factors, like naturally occurred in ECM. For example, increase collagen stiffness by crosslinking is always associated with reduced interstitial space for nutrients and metabolites diffusion. It is impractical to separate these factors to study but we should be very careful when we interpret results.

It has been proposed that the ECM is a critical regulator of cellular dormancy [6]; however, the role of matrix restriction in regulating this process has not been specifically addressed. We have demonstrated that tumor cells can enter and exit dormant conditions by modulating the matrix physical property through autocrine and paracrine enzymatic action. We also showed that the cell states of cancer cells not only have “On/Off” states switched to fast growing or dormancy, but other states in between. Physiologically, the modification of tumor microenvironment could be also contributed by inflammatory cells, fibroblasts or other stroma cells in the system. Disseminated cancer cells usually lodge in a non-orthotropic tissue microenvironment and the current state of cell–matrix interaction determine the cell fate of these tumor cells [39]. Indeed, The expression and activity of MMPs are increased in almost every type of human cancer, and this correlates with advanced tumor stage, increased invasion and metastasis, and shortened survival [12, 13]. Tumor recurrence always happens at the incision scar wound sites where the fibrous tissue wraps the tumor cell to render them quiescent for a period of time [40]. Therefore, reciprocal interactions of the microenvironment and tumor cells might decide the cell fate for proliferation or growth arrest. Tumor dormancy is commonly observed clinically. Dormant tumor cells can avoid clinical detection and escape aggressive chemo therapy by hiding in protective microenvironments either inside tumor or in specific organs. In many cancers, there are late relapses after years or decades of complete remission [17–19]. Research on the dormant stage of cancer has been limited due to the lack of appropriate models. Our competent/dormant models may provide a toolbox to study this population of cells and to identify regulators of the switch, hence to assist the therapeutic target identification.

Matrix stiffness has substantial impact upon drug response. In the 3D cell culture model, we observed the significant environmental impact on reducing drug efficacy. Mechanical restriction induced drug resistance could result from slow metabolic rate for drug uptake or low membrane permeability hindering drug penetration. In addition, other mechanisms could possibly facilitate cancer cells to survive, such as tumor cells assembling into tumoroids to tolerate drug toxicity [41–43], activation of multidrug resistance protein to export drugs out of cells [44, 45], abnormal reduction potential to metabolize drugs in unique pathways [46, 47], or the summation of all possibilities listed above. As we observed, MDA-MB-231reducing potential as of CCK reading was increased instead of decrease after 72-hour paclitaxel treatment compared to controls (Fig. 5c), demonstrating that cells tend to build a compensationally new redox balance with oxidative insults. Intriguingly, we also have been able to demonstrate that cells surviving drug treatments can regain competent growth in vitro and form tumor in vivo. In line with these observation, clinical data shows that drug resistance nearly always develops in slow cycling cells [48]. Therefore, an important question is raised of how to target these quiescent cells? currently, most chemo drugs target cell cycling inhibition, few models have developed to test chemo drug on slow growing cells. Col-Tgel 3D gel systems, including the same cell type in different proliferation co, conditions serve as a practical model to test drug efficiency.

## Conclusion

We have developed a *physiologically relevant 3D* platform for the study of tumor biology and drug response during tumor cell proliferation states, from competency to dormancy. It contains three phases, tumoroid formation, drug treatment, and cellular analysis; and it can be easily modified based on cell type specific requirement. While using 3D tunable matrices, we can setup cell type specific growth profiles for cell behavior studies. The programs are able to simulate clinical regimens in forms of single, repeated, or combination treatments with defined intervals for cell recovery. Cellular activities, such as alterations of cell morphology, marker genes/proteins expression, metabolism rates, mobility and mortality can be studied from culture media, cells in situ, or released cells. This experimental setup is practical for achieving a high throughput drug test.
